# Zoonotic nematode larvae in Greenland halibut (*Reinhardtius hippoglossoides*) from Greenlandic waters: Occurrence, fillet distribution and association with Mushy halibut syndrome

**DOI:** 10.1016/j.ijppaw.2025.101181

**Published:** 2025-12-24

**Authors:** Natacha L. Severin, Andrea K. Bisbjerg, Kitt U. Ristinge, Kaan Kumas, Liliana I. Ferrão, Per W. Kania, Kurt Buchmann

**Affiliations:** a Laboratory of Aquatic Pathobiology (AQUA), Section for Parasitology and Pathobiology, Department of Veterinary and Animal Sciences, Faculty of Health and Medical Sciences, University of Copenhagen, Stigbøjlen 7, 1870, Frederiksberg C, Denmark; b Department of Animal Sciences and Aquatic Ecology, Ghent University, Coupure Links 653 – block F, B-9000, Gent, Belgium

**Keywords:** Fish parasitology, Mushy halibut syndrome, Greenland halibut, *Anisakis simplex*, *Contracaecum osculatum*, *Phocanema bulbosa*

## Abstract

Zoonotic anisakid nematodes commonly infect wild fish, and their presence in commercially caught species represents a consumer hazard due to risk of anisakidosis. The Greenland halibut (GLH) is a species of high commercial value to many countries, including Kalaallit Nunaat (Greenland), where GLH is the second most exported marine species. Despite this, the occurrence of anisakid nematodes in the flesh of GLH remains poorly studied. Additionally, the relationship between anisakid infection and ‘Mushy halibut syndrome’ (MHS), a condition affecting the fillet quality of GLH, has not been investigated. The aim of this study was to extend our knowledge of the occurrence of anisakid nematodes in GLH, with emphasis on fillet distribution, and explore associations with MHS. GLH (n = 104) from three offshore fishing grounds underwent necropsy and parasitological examination of the main fillet and belly flaps. Overall prevalence of third-stage larval infection was 44.2 %, while mean intensity and abundance were 4.6 and 1.3, respectively. When examining the fillets, third-stage larvae were predominantly found in the belly flaps. For a subsample of fish (n = 55), the investigation included parasitological examination of the body cavity and liver as well as extended necropsy, quality index method assessment and age estimation. The isolated third-stage larvae belonged to three species: *Anisakis simplex* s. s. was most common (n = 721), followed by *Contracaecum osculatum* (n = 36). *Phocanema bulbosa* was found in the body cavity and liver only (n = 11). MHS was associated with higher infection levels in the belly flaps and liver, lower condition factor and higher gonadosomatic index. The results presented here have implications for wild fish stock monitoring and food safety. Recording of the parasite prevalence and distribution in GLH can assist risk management and provide information on the status of the fish species in the ecosystem.

## Highlights

• This study mapped the distribution of anisakid larvae in Greenland halibut.
• *Anisakis simplex*, *Contracaecum osculatum and Phocanema bulbosa* were identified.
• *A. simplex* s. s. dominated in the fillet and occurred mainly in the belly flaps.
• Belly flap and liver parasitism were associated with Mushy Halibut Syndrome.
• Parasitological investigations support ecosystem and food safety assessments.

## Graphical abstract

**Figure:**
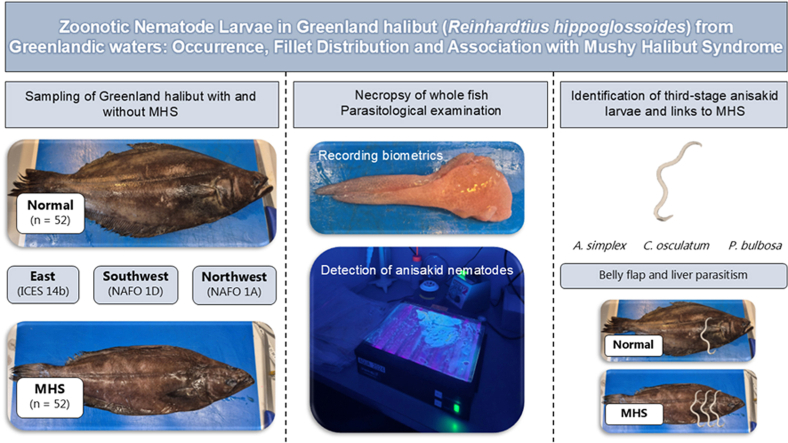

## 1 Introduction

The Greenland halibut (*Reinhardtius hippoglossoides*) is a benthopelagic flatfish belonging to the family *Pleuronectidae*. It inhabits the Arctic, North Atlantic and North Pacific Ocean at depths of 200 to >2000 m and temperatures between −1 °C and 6 °C (Jørgensen, 1997a; Vis et al., 1997; Boje and Hjörleifsson, 2000; Bowering and Nedreeas, 2000; Boje et al., 2014; Orlova et al., 2017). The diet of Greenland halibut is opportunistic, with an ontogenic shift in preferred main food sources, and the species is prey to a number of predatory marine mammals including narwhales (*Monodon monoceros*) (Laidre et al., 2004; Laidre and Heide-Jørgensen, 2005), harp seals (*Pagophilus groenlandicus*) (Kapel, 1995; Hammill and Stenson, 2000) and hooded seals (*Cystophora cristata*) (Folkow and Blix, 1999; Haug et al., 2007).

Greenland halibut is of commercial interest to several countries including Kalaallit Nunaat (Greenland). Greenland is the world's largest supplier of Greenland halibut products, and every year, catches exceeding 33.000 tons are distributed fresh-frozen or processed into convenience products for the global market (Karl et al., 2018; Naatsorsueqqissaartarfik, 2024; RG, 2025). The commercial fishery for Greenland halibut takes place inshore and offshore, and important fishing grounds are assigned to regions, with each of these management units considered a separate stock complex (Long and Jones, 2021; Vihtakari et al., 2022). The species is of paramount importance to Greenland's economy (Siunnersuisoqatigiit, 2020; Naatsorsueqqissaartarfik, 2022), and holds high cultural significance with respects to the indigenous Greenlandic diet (*kalaalimerngit*) (Hauptmann, 2024): Greenland halibut meat has traditionally been prepared in different ways to ensure taste and food security, and preservation methods such as drying and pickling are still practiced in today. Dried halibut is an expensive delicacy and pickled or conserved halibut is typically enjoyed on special occasions. In the North, halibut meat is also enjoyed raw or frozen as a snack, and raw, frozen or processed leftovers are used to feed Greenland sled dogs (*Canis lupus familiaris borealis*) (VFMG and Veterinær- og Fødevaremyndigheden i Grønland, 2019; Reimer-Johansen, 2024).

Despite the commercial and cultural value of Greenland halibut, the prevalence and distribution of zoonotic nematodes in the fillet meat is scarcely investigated. In general, publications on zoonotic nematodes in the fillets of commercially important fish species from Greenlandic waters are limited and of older date (Platt, 1975, 1976; Boje et al., 1997). Zoonotic parasites can be indicators of ecosystem health, including effects of climate change. Climate change is of particular concern in the Arctic for many reasons, one being increased risk of zoonotic infections including human anisakidosis (Bard, 1999; ACIA, 2004; Burek et al., 2008). Human anisakidosis is a food-borne zoonotic disease induced by ingestion of live zoonotic third-stage nematode larvae belonging to the *Anisakidae* family. These include the genera *Anisakis*, *Phocanema* and *Contracaecum*. The disease is characterized by gastrointestinal symptoms, and in severe cases anaphylaxis (Bao et al., 2019).

The extent of anisakidosis in Arctic regions is poorly elucidated (Parkinson et al., 2014; Sonne et al., 2020). The current recommendations by the European Food Safety Authority (EFSA) to avoid infection is to adequately pre-treat fish products (EFSA et al., 2024). To kill *Anisakis* larvae, EFSA recommends that fish products intended for raw or almost raw consumption are kept frozen (core temperature) at −15 °C for at least 96 h, −20 °C for at least 24 h, or at −35 °C for at least 15 h. Heat treatment of the fish products with parasites should secure a core temperature of minimum 60 °C for at least 1 min for killing of *Anisakis* larvae (EP, 2004; EFSA, 2010; EFSA et al., 2024). While the consumption of fish prepared according to traditional indigenous food practices may pose a risk of contracting anisakidosis due to insufficient parasite inactivation (Pufall, 2010; Davidson et al., 2011; Pufall et al., 2012; Hauptmann et al., 2020), population-based information concerning its occurrence and extent is lacking in most remote communities (Møller et al., 2007; Rokicki, 2009; Goyette et al., 2014).

In addition to health concerns, some parasites are known to be able to adversely affect the fillet quality of commercial fish species. Among these are the species *Kudoa* and *Myxobolus*, but even anisakid nematode larvae can impact fillet quality (Mohamed et al., 2020; Ryberg et al., 2020). ‘Mushy halibut syndrome’ (MHS) is a condition occurring in Greenland halibut characterized by soft and jelly-like fillets with inferior texture, flavor and low yield. No cause of the syndrome has been identified, and although it is generally considered a non-infectious myopathy, its association with *Anisakidae* infection has not been investigated (Severin and Buchmann, 2024; Severin et al., 2025b).

The aim of this study was to extend our knowledge of the distribution and prevalence of zoonotic anisakid nematode larvae in the main fillet and belly flaps, as well as body cavity and liver, of Greenland halibut, and investigate any association between geography, host biometrics or fillet quality (MHS) and parasite burden.

## 2 Materials and methods

### 2.1 Sampling and MHS evaluation

A total of 104 Greenland halibut caught in three different fishing grounds were used in the current study. 29 fish were from Southwest Greenland (“SW”, NAFO Subareas 1C/1D), 13 fish were from East Greenland (“E”, ICES 14b) and 62 fish were from Northwest Greenland (“NW”, NAFO Subarea 1A) were sampled and labelled as normal or MHS during routine quality inspection by trained quality professionals working in the fisheries using the following approach: Fish were sampled aboard the vessel immediately after capture and MHS status was assessed based on two criteria: 1) decreased muscle firmness and elasticity upon application of manual pressure to the musculature (finger force), 2) edematous feel upon palpation of musculature and in severe cases musculature quivering to the touch. The normal references were identified using the same approach, but with reverse criteria concerning muscle firmness and elasticity. To ensure sample homogeneity, the normal references were selected to match the size of the MHS fish samples.

All fish were caught by large commercial Royal Greenland factory trawlers using Vónin bottom trawls and stored at −20 °C. Bottom temperatures ranged from 0.5 to 1.4 °C (average 1 °C). All registered biometric data are available in Supplementary material 1.

### 2.2 Necropsy

All thawed fish underwent qualitative exterior inspection and necropsy to record fish length, weight and condition, sex and reproductive status, gonad, liver and fillet weight, parasite burden and symptoms of underlying pathology using the standardized protocol found in Supplementary material 2. The body musculature (the fillets) was removed and de-skinned with a filleting knife and prepared for parasitological examination as described in subsection 2.3. Gonadal maturity staging was carried out following an approach similar to the one described by (Albert et al., 2001).

In addition to necropsy and parasitological examination of main fillet and belly flaps, a subsample of fish underwent quality index method assessment (QIM) and parasitological inspection of body cavity, heart and liver. Furthermore, age was estimated by otolith reading, and stomach fullness, gallbladder fullness and bile color was recorded to investigate a relationship between age, feeding status and parasite burden. Details are contained in Supplementary material 2.

After freezing and transport to the University of Copenhagen, Denmark, freshness and quality of the thawed samples was assessed using the QIM scheme for thawed whole Greenland halibut developed by López-García et al. (2014). QIM was applied to document possible variations in product quality between fish affected and unaffected by MHS that may help identify affected fish and assist commercial product categorization.

The stomach was excised and its content was evaluated using a scale from 0 (empty) to full (4) (Mjanger et al., 2020). The gallbladder and bile duct were separated from the liver, and gallbladder fullness and bile color was assessed (Karinen et al., 2010).

Otoliths were removed, rinsed, labelled and stored dry in small paper envelopes. Age estimation was performed by counting annuli on the surface of the whole left otolith. If the left otolith was damaged, the right otolith was used. An annulus was defined as a pair of translucent and opaque zones (Albert et al., 2009). Prior to reading, the otoliths were immersed in deionized water for 24 h to improve readability, and reading was performed while the otoliths were still submerged (Gregg et al., 2006; Karlson et al., 2012).

### 2.3 Parasitological examination

The main fillet and belly flaps underwent macroscopical inspection, hydraulic compression (Bacho Hydraulic compression tool 15T BH715, Sweden) and UV-examination for detection of parasitic nematodes (Zuo et al., 2016; ISO, 2021). For the subsample of fish that underwent additional examination, body cavity, heart, gallbladder and liver were also inspected.

To record the spatial distribution of parasites, belly flaps were isolated and the main fillet were categorized as dorsal right, dorsal left, ventral right and ventral left, and each fillet was divided into an anterior, medial and posterior part, before being subjected to hydraulic compression.

All recovered parasites were preserved in 96 % ethanol and prepared for morphological and molecular identification following the protocol described by Mehrdana et al. (2014).

Supplementary material 3 contains the raw data on parasite distribution in the investigated organs, between geographical locations and between length categories.

#### 2.3.1 Morphological identification

The anterior and caudal parts of the nematodes were excised, cleared in lactic acid and mounted on microscope slides in DPX (DPX Mountant for histology, cat.no. 06522, Merck Life Science ApS, Denmark) or Aquatec® (Merck, Brøndby, Denmark), and examined under a light microscope (Leica DM 5000 B; Leica, Wetzlar, Germany). The specimens were examined and categorized according to genus specific morphological characteristics, as described by Val'ter et al. (Val'ter et al., 1982).

#### 2.3.2 Molecular identification

The mid-sections of the recovered nematodes were used for PCR and subsequent gene sequencing. PCR was targeting ribosomal DNA (rDNA) and mitochondrial DNA (mtDNA). Thus, sequencing of the internal transcribed spacer (ITS) region from 18S to 28S through ITS1, 5.8S, and ITS2, and the mitochondrial gene cytochrome *c* oxidase subunit 2 (*cox2*) were used for molecular species identification.

The mid-sections of the recovered nematodes were used for DNA purification by the means of QIAamp DNA Mini Kit (cat. No. 61306, Qiagen, Denmark) according to manufacturer's instructions (except that the elution buffer volume was 50 μl instead of 200 μl).

PCR was conducted in a Thermal Cycler BioRad T100 (BioRad, Denmark) using 60 μL reaction volumes consisting of 6 μL of 10 × Reaction buffer, 1.5 mM MgCl_2_, 3 units of DNA Polymerase, (all three BIOTAQ DNA Polymerase, Nordic BioSite, Denmark), 1 mM dNTPmix (Applied Biosystems™ GeneAmp™ dNTP Blend (100 mM), Fisher Scientific, Denmark), forward primer (1 mM), and reverse prime r(1 mM) (both from Tag Copenhagen, Denmark) 6 μL of sample and, finally UltraPure™ DNase/RNase-Free Distilled Water (Fisher Scientific, Denmark) up to 60 μl.

The general PCR conditions were pre-denaturation at 95 °C for 5 min, 40 amplification cycles of denaturation at 95 °C for 30 s, annealing at primer specific temperature for 30 s, elongating at 72 °C (approximately 1 min/1000 bp) followed by post-elongation at 72 °C for 7 min. Primer combinations, target regions and specific PCR conditions are presented in Table 1. Further details concerning Primer sequences and references can be found in Supplementary material 4.

**Table 1:** PCR primer combinations, targets, annealing temperatures (Ta) and elongation time.

PCR primer combinations		Target site	Specific PCR conditions	
Forward	Reverse		Ta	Elongation time
PDG_18S_F5	NC2	18S - ITS1 - 5.8S - ITS2	54 °C	2 min
211F	210R	*cox2*	Td 53->46 °C	30 s
211F_alt	210R	*cox2*	55 °C	30 s
211F_alt	CoOs_Mith_R3	*cox2*	55 °C	45 s

The PCR products were visualized by 2 % agarose gel electrophoresis and purified using the Illustra™ GFX™ PCR and Gel Band Purification Kit (VWR, Denmark). Sequencing was conducted at Macrogen Europe (Netherlands) and analyzed on the CLC Main Workbench v20.0.4 (Qiagen, Aarhus, Denmark) by BLAST® (Bethesda, Maryland, USA). The obtained sequences were submitted to GenBank.

The obtained sequences were subjected to phylogenetic analysis to identify samples to species level. The alignments were performed by CLUSTAL W using the entire obtained sequences (Thompson et al., 1994). The rDNA was trimmed to consist of the ITS1 (5.8s) and ITS 2-regions, which means that the 3′ ends of 18s and 5’ ends of 28S were trimmed off. The *cox2* gene was trimmed to consist of the part between the forward primer 211F or 211F_alt, and the reverse primer 210R. Model testing revealed in both cases that GTR + G + T was the best evolutionary model. Maximum Likelihood phylogenies were performed using 1000 bootstraps and Unweighted Pair Group Method with Arithmetic Mean (UPGMA) starting trees.

## 3 Statistical analysis

Infection prevalence, mean intensity and abundance for the entire sample were calculated (Bush et al., 1997). In addition, the mean number of detected parasites was calculated for each examined fillet part, as well as for the body cavity and liver in the subsample.

The normality and lognormality of data was assessed by Shapiro–Wilk normality test. Grubb's test was used to identify outliers. Variance between study groups and categories was investigated using either non-parametric or parametric tests. Non-parametric data were evaluated using either Kruskal-Wallis test followed by a Dunn's correction for multiple comparisons post hoc (*H*-value, *df* and *p*-value reported), or Mann-Whitney *U* test (*U-*value and *p*-value reported). Parametric data were evaluated using Unpaired *t*-test (*t*-value, *df* and *p*-value reported). Spearman's rank correlation coefficient (ρ) was used to evaluate the association between parasite burden, biometric variables and MHS (ρ-value reported and *p*-value reported).

All statistical analyses were conducted using GraphPad Prism for Windows (GraphPad Software, Version 10.2.3 (403), Boston, Massachusetts USA, www.graphpad.com) with a probability level of 5 % (α) and a 95 % confidence interval (CI).

Condition factor (Fulton's *K*), hepatosomatic index (HSI) and gonadosomatic index (GSI) were calculated for all fish using the formulae below.

$$K=\frac{W}{L^{3}}\times 100$$

$$HSI=\frac{W_{L}}{W}\times 100$$

$$GSI=\frac{W_{G}}{W}\times 100$$

where.

L = Total fish length in cm.

W = Total fish weight in g.

W_L_ = Liver weight in g.

W_G_ = Gonad weight in g.

## 4 Results

### 4.1 Parasite-host relationships

Total fish length and weight were in the span of 36.5–89.5 cm and 290–8115 g, respectively. The sample represented an equal distribution of males and females, as well as fish with and without signs of MHS, as shown in Table 2.

**Table 2:** Host biometrics of 104 Greenland halibut. Total fish length, total fish weight, condition factor (Fulton's *K*), HSI and GSI are given as mean ± SD (range).

Parameter	E	SW	NW	Total
**No. examined fish**	13	29	62	104
**Sex distribution (male/female)**	8/5	21/8	23/38^a^	52/51^a^
**Total length (cm)**	62.5 ± 11.6 (49–82)	51.3 ± 3.5 (45–59)	54 ± 11 (36–89.5)	54 ± 10 (36–89.5)
**Total fish weight (kg)**	1983 ± 1250 (692-3916)	982.6 ± 216.3 (318-1412)	1538 ± 1310 (290-8115)	1443 ± 1144.6 (290-8115)
**Condition factor (*K*)**	0.8 ± 0.6 (0.63–1.1)	0.8 ± 0.1 (0.2–0.9)	0.2 ± 0.1 (0.5–1.3)	0.79 ± 0.1 (0.2–1.3)
**HSI**	1.1 ± 0.5 (0.3–2.2)	1.1 ± 0.7 (0.4–3.4)	1.6 ± 1.6 (0.4–9.6)^b^	1.4 ± 1.3 (0.3–9.6)^b^
**GSI**	1.8 ± 1.3 (0.2–3.8)	2.8 ± 2.3 (0.2–8.3)	0.9 ± 0.9 (0.03–3.5)	1.5 ± 1.7 (0.03–8.3)
**MHS status (normal/MHS)**	7/6	13/16	32/30	52/52

a The sex of one fish was indeterminable.

b HSI could not be calculated for two fish.

A total of 768 third-stage anisakid nematode larvae (henceforth ‘third-stage larvae’) were isolated from the main fillet, belly flaps, body cavity and liver of all examined fish. No larvae were detected in any examined hearts. The identified larvae belonged to three species: *A. simplex* s. s., *C. osculatum* and *P. bulbosa*. The latter was only detected in the body cavity and liver of four fish.

A total of 277 third-stage larvae were isolated from the main fillet and belly flaps of 46 infected fish (n = 104). Overall infection prevalence was 44.2 %, while mean intensity and abundance were 4.6 and 1.3, respectively. Four fish accounted for the majority of third-stage larvae found, with 76, 20, 15 and 10 larvae isolated from their belly flaps, respectively. Three of these fish were from SW, while one was caught in E. Overdispersion was expected, and we identified one sample in the MHS group as an outlier (the sample named SW_J3 in Supplementary material 1, although this sample was not removed during subsequent data analysis to prevent omitting important information.

**Fig. 1:**
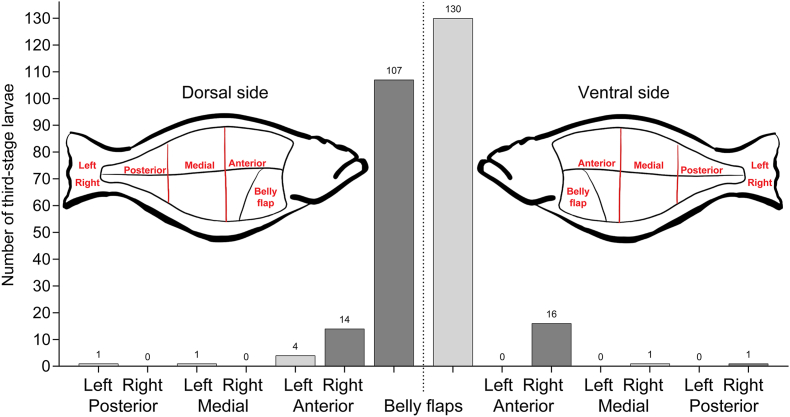
Distribution of third-stage anisakid nematode larvae in dorsal and ventral muscle sections of 46 infected Greenland halibut.

When examining fillet distribution, third-stage larvae were predominantly found in the belly flaps compared to the main fillet parts, as illustrated in Fig. 1. The mean number of larvae isolated from the main fillet was 2.1, compared to 5.7 larvae for the belly flaps. Most of the larvae isolated from the main fillet and belly flaps were identified as belonging to the genus *Anisakis* (n = 270) based on morphological characteristics, with a smaller number of *Contracaecum osculatum* larvae (n = 7). Details on infection parameters relating to each fillet part examined and each larval identified species are shown in Table 3. The 29 fish caught in SW displayed the highest parasite load with a total of 24 third-stage larvae isolated from the main fillet and 150 from the belly flaps. In comparison, the 62 fish from NW carried only 6 third-stage larvae in the main fillet and 36 in the belly flaps. Similar results were obtained from the 13 fish caught in E, which harbored a total of 8 third-stage larvae in the main fillet and 53 in the belly flaps.

**Table 3:** Infection parameters of *A. simplex* s. s. and *C. osculatum* recorded in main fillet (MF) and belly flaps (BF) of 104 Greenland halibut from three Greenlandic fishing grounds. *Mean no. larvae* reflects the mean number of isolated third-stage larvae per fillet part of each infected fish.

Infection parameters	Location	*A. simplex* s. s.				*C. osculatum*			
		E	SW	NW	Total	E	SW	NW	Total
No. infected fish	*MF*	5	8	5	18	0	0	0	0
	*BF*	11	16	13	40	1	2	1	4

No. larvae in total	*MF*	8	24	6	38	0	0	0	0
	*BF*	52	145	35	232	1	5	1	7

Range	*MF*	0–3	0–13	0–2	0–13	N/A	N/A	N/A	0–7
	*BF*	0–15	0–76	0–7	0–76	0–1	0–4	0–1	0–4

Mean no. larvae	*MF*	1.6	3	1.2	2.1	N/A	N/A	N/A	N/A
	*BF*	4.7	9	2.7	5.8	1	2.5	1	1.8

N/A = Not applicable.

Analysis by two-tailed Mann-Whitney *U* test revealed no significant differences in parasite burden between the sexes (*U* = 406, *p*-value = 0.69). Moreover, no differences were found when assigning fish into three length categories (0–49 cm; 50–59 cm; 60–90 cm) and comparing them using a two-tailed Kruskal-Wallis test followed by Dunn's multiple comparisons test (*H* = 2.7, *df* = 2, *p*-value = 0.26).

A total of 491 third-stage larvae were isolated from the liver (n = 276) and body cavity (n = 215) of 38 infected fish (n = 55). The mean number of isolated larvae was 16.5 for the body cavity, and 7.9 for the liver. Table 4 shows the infection parameters for the different areas sampled and for each identified anisakid species. Again, five fish accounted for the majority of third-stage larvae found, with 26, 40, 25, 22 and 30 larvae isolated from their livers, and 23, 31, 35, 17 and 72 larvae isolated from the body cavity. Four of these fish were from E, while one was caught in SW. The infection burden of males (n = 30) and females (n = 25) was compared using a two-tailed Mann-Whitney *U* test, and no significant differences were found between the sexes (*U* = 189, *p*-value = 0.07).

**Table 4:** Infection parameters of *A. simplex* s. s., *C. osculatum* and *P. bulbosa* recorded in the body cavity (BC) and liver of 55 Greenland halibut from three Greenlandic fishing grounds. *Mean no. larvae* reflects the mean number of isolated third-stage larvae from the body cavity and liver of each infected fish.

Infection parameters	Location	*A. simplex* s. s.				*C. osculatum*				*P. bulbosa*			
		E	SW	NW	Total	E	SW	NW	Total	E	SW	NW	Total
No. infected fish	*BC*	5	4	4	13	2	1	2	5	0	0	0	0
	*Liver*	10	11	13	34	5	5	5	15	1	1	1	3

No. larvae in total	*BC*	107	86	9	202	4	1	2	7	6	0	0	6
	*Liver*	130	66	53	249	5	11	6	22	2	0	3	5

Range	*BC*	0–35	0–71	0–4	0–71	0–3	0–1	0–1	0–3	0–6	N/A	N/A	0–6
	*Liver*	0–39	0–30	0–18	0–39	0–1	0–3	0–2	0–3	0–2	N/A	0–2	0–2

Mean no. larvae	*BC*	21.4	21.5	2.3	15.5	2	1	1	1.4	6	N/A	N/A	0.8
	*Liver*	13.0	6	4.4	7.5	1	1.8	1.2	1.4	2	N/A	1.5	1.7

N/A = Not applicable.

Spearman's rank coefficient (ρ) was calculated to investigate the association between parasite burden and biometric variables as well as MHS status. One correlation matrix was generated for the entire sample (n = 104), and an additional one for the subsample of fish that underwent extended examination (n = 55), where age, gall bladder fullness, bile color, QIM score were included in the matrix. MHS status was expressed as ordinal values, where 0 = Normal (no MHS) and 1 = MHS.

**Fig. 2:**
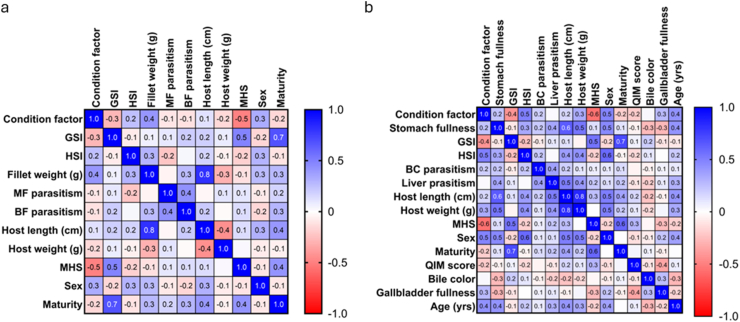
Heat maps generated using a two-tailed Spearman's correlation test (*p* < 0.05 and *df* = 104-2 (a); 55-2 (b)). **a** Correlation analysis of biometric and parasitological data obtained from 104 Greenland halibut **b** Correlation analysis of biometric and parasitological data obtained from the subsample of 55 Greenland halibut. Spearman's rho (ρ) defines the strength and direction of the relationship between the variables in the matrix. Stronger colors with values closer to 1 or -1 indicate a strong relationship between the variables, while lighter colors with values closer to 0 indicate a weak relationship. *MF* = Main fillet; *BF* = Belly flaps; *BC* = Body cavity.

In the first matrix, we found no significant correlation between parasite burden and any other parameters, as illustrated in Fig. 2a. The second matrix revealed a moderate positive relationship between liver parasitism and host length (ρ = 0.5, *p*-value = <0.0001) and weight (ρ = 0.4, *p*-value = 0.002). When the parasite burden documented in the main fillet and belly flaps was factored into the equation, belly flap parasitism showed a moderate positive correlation with liver parasitism (ρ = 0.51, *p*-value = <0.0001).

### 4.2 Associations between parasite burden, host biometrics and MHS

The total sample of 104 Greenland halibut represented 52 fish with signs of MHS and 52 normal specimens. Twenty-six normal fish and 24 fish affected by MHS carried third-stage larvae in the main fillet and belly flaps. As for the subsample of 55 fish, infection in the body cavity and liver was recorded in 19 normal fish and 19 fish affected by MHS. Details for each of the two groups are depicted in Table 5.

**Table 5:** Number of third-stage larvae detected in the main fillet (MF), belly flaps (BF), body cavity (BC) and liver of Greenland halibut affected and unaffected by MHS.

Sample size (n)	Examined organ	MHS		Normal	
		No. infected fish	No. larvae in total	No. infected fish	No. larvae in total
104	*BF*	23	189	19	50
	*MF*	11	27	7	11

55	*BC*	7	148	6	67
	*Liver*	18	191	17	85

The parasite burden between the two groups was compared by two-tailed Mann-Whitney *U* test, and the analysis showed that fish affected by MHS harbored a significantly higher number of third-stage larvae in the belly flaps (*U* = 118, *p*-value = 0.02) and liver (*U* = 79.5, *p*-value = 0.02) compared to normal fish.

Differences in maturity status were assessed using Unpaired *t*-test, and analysis revealed a statistically significant difference between normal fish and fish affected by MHS, the MHS-group representing a higher amount of mature and spawning individuals (*t* = 4.6, *df* = 101, *p*-value = <0.0001). Further comparisons between the two groups were conducted using the Mann-Whitney *U* test. The analyses showed that normal fish exhibited significantly higher *K*-values (*U* = 578.5, *p*-value = <0.0001), while fish affected by MHS had higher GSI-values (*U* = 597.5, *p*-value = <0.0001). This is supported by the findings from the correlation matrix presented in Fig. 2a, which shows a moderate negative correlation between condition factor and MHS (ρ = −0.5, *p*-value = <0.0001), and a moderate positive correlation between MHS and GSI (ρ = 0.5, *p*-value = <0.0001), and MHS and maturity (ρ = 0.4, *p*-value = <0.0001).

Analysis of the data from the subsample of fish that underwent additional examination revealed a statistically significant difference in QIM score between normal fish and fish affected by MHS, normal fish exhibiting an overall lower score reflecting better quality (*U* = 252.5, *p*-value = 0.03). The two groups also differed in gall bladder fullness, normal fish generally having fuller gall bladders during necropsy (*U* = 276.5, *p*-value = 0.04). MHS status was not influenced by sex (*U* = 315.5, *p*-value = 0.28). Except for one fish, which exhibited extreme organ degeneration, necropsy did not reveal any signs of disease consistent with MHS status. The fish that underwent additional examination were estimated to be between 4 and 17 years of age (average age 8.6), and we did not find age to differ significantly between normal fish and fish affected by MHS (*U* = 297.5, *p*-value = 0.17). Further details concerning the correlation analysis can be found in Supplementary material 5.

### 4.3 Morphological identification

Three species were identified across both studies, and all belonged to the family *Anisakidae*. Most of the larvae were identified as *Anisakis* sp. (n = 721) based on morphological characteristics, with a smaller number of *Contracaecum* spp. (n = 36) and *Phocanema* sp. (n = 11). *Anisakis* spp. was found in all examined organs, while *Contracaecum* spp. was only present in belly flaps, body cavity and liver. *Phocanema* sp. was only isolated from the body cavity of one fish and the liver of three fish.

### 4.4 Molecular identification

A subsample of 68 third-stage larvae was selected for molecular identification. The sequences of some of the specimens within one species designation had 100 % identity to each others; only one specimen from each of these groups with 100 % identity was used together with the unique ones in the phylogenetic analysis. Details of the result of sequencing, indication of grouping and GenBank accession numbers are included in Supplementary material 4.

**Fig. 3:**
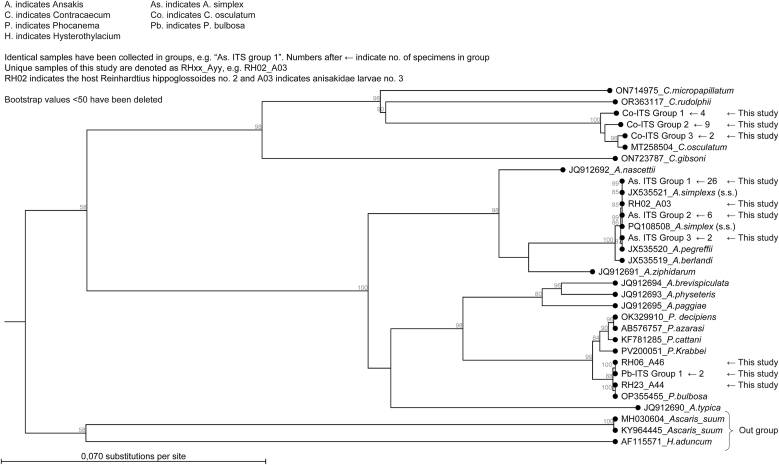
Phylogram illustrating the evolutionary relationships among anisakid nematode larvae inferred from ITS-region sequences. Identical samples were grouped (e.g., *As. ITS Group 1*), with the number of specimens indicated after the group name. Samples generated in this study are marked with arrows, and host information is provided. Outgroups include *Ascaris suum* and *Hysterothylacium aduncum*. The tree was constructed using the Maximum Likelihood method with CLC Main Workbench v20.0.4 (Qiagen, Aarhus, Denmark) under the GTR + G + T evolutionary model.

**Fig. 4:**
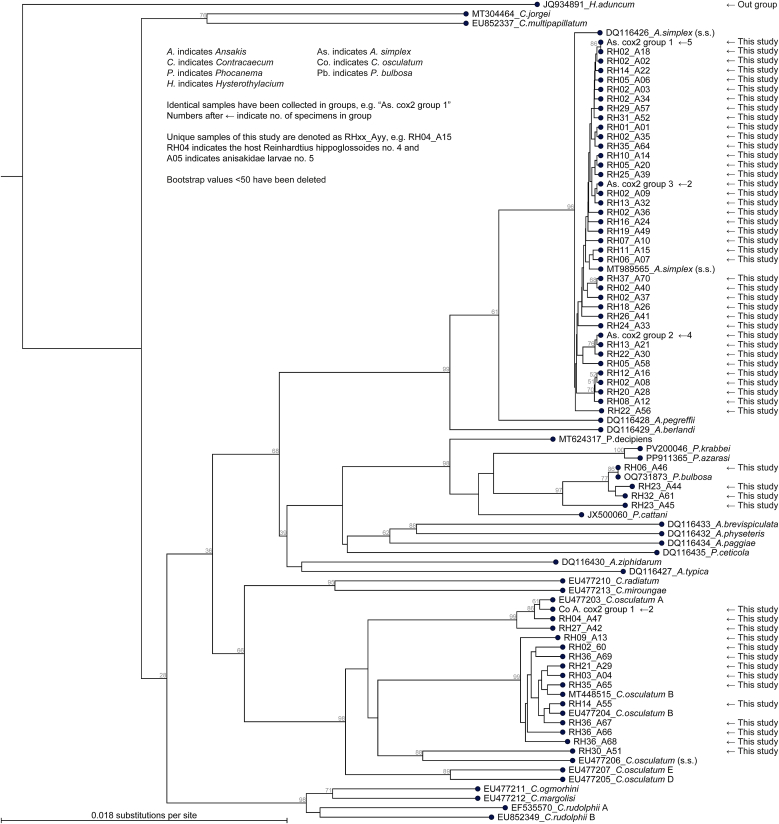
Phylogram depicting the evolutionary relationships among anisakid nematode larvae inferred from mitochondrial cox2 gene sequences. Identical samples collected in groups are indicated, e.g., “*As. cox2 group 1*”, with the number of specimens in each group shown after the group name. Unique samples generated in this study are labelled with host codes and larval sample id. Reference sequences from GenBank are denoted with accession numbers. Outgroups include *Hysterothylacium aduncum* and *Contracaecum multipapillatum*. The tree was constructed using the Maximum Likelihood method implemented in CLC Main Workbench v20.0.4 (Qiagen, Aarhus, Denmark) under the GTR + G + T evolutionary model.

Preliminary molecular identification was achieved by BLAST searches at National Center for Biotechnology Information (NCBI, USA), and more precise identifications were obtained by phylogenetic analysis. These are presented as phylograms in Fig. 3 (ITS-region) and Fig. 4 (cox2).

The molecular approaches were consistent with each other and with morphological identification (section 4.3). 49, 15, and 4 specimens were identified as *A. simplex* s. s., *C. osculatum*, and *P. bulbosa*, respectively. One specimen was identified as *C. osculatum* B based on *cox2*. One specimen produced no ITS-region product; this specimen was identified as *C. osculatum* s. s. based on *cox2*. Two specimens produced no *cox2* products; these two specimens were identified as *A. simplex* based on the ITS-region.

#### 4.4.1 A. simplex s. s

With respect to the ITS region, they were represented by three groups consisting of 41, 5, and 2 specimens together with one unique specimen. With respect to *cox2*, they were represented by 3 groups consisting of 5, 4, and 2 specimens together with 36 unique specimens. All specimens grouped together with *A. simplex s.s.* upon phylogenetic analysis (Fig. 3).

#### 4.4.2 C. osculatum

With respect to the ITS region, they were represented by two groups consisting of 9 and 4 together with 1 unique specimen.

With respect to *cox2* all sequences were quite diversified. However, when subjected to BLAST at GenBank, they were represented by 3 groups consisting of 10 *C. osculatum* B, 4 *C. osculatum* A, and 1 *C. osculatum* s. s. Two of the specimens in the group of *C. osculatum* A were identical, the remaining 2 were unique. All 10 *C. osculatum* B were unique. The result based on *cox2* was consistent with the grouping based on the ITS-region; *C. osculatum* group 1, *C. osculatum* group 2, and the unique from the ITS-region phylograms corresponded to *C. osculatum* A, *C. osculatum* B, and *C. osculatum* s.s. from the *cox2* phylograms, respectively.

#### 4.4.3 P. bulbosa

With respect to the ITS region, 2 specimens were identical, and 2 specimens were unique. However, identification as *P. bulbosa* was not certain upon BLAST, but was supported by the phylogenetic analysis (Fig. 3). With respect to *cox2*, all sequences were unique and could be identified as *P. bulbosa* upon BLAST as well as phylogenetic analysis (Fig. 4).

## 5 Discussion

### 5.1 Parasite-host relationships

Most of the parasites isolated from the fillets were located in the belly flaps. This is likely because the belly flaps are the nearest location after the larvae penetrate and migrate to the musculature following ingestion by the host (Smith and Wootten, 1975), but may also be attributable to a more suitable lipid content, tissue thickness and blood supply compared to other parts of the fish's musculature (Strømnes, 2014).

Previous studies on Greenland halibut from the Northwest and Northeast Atlantic Ocean and the Barents Sea have found the species to host *A. simplex*, *P. decipiens*, *C. osculatum* and *Hysterothylacium aduncum* (Poljanskij, 1955; Wierzbicka, 1991a, 1991b, 1992; Arthur and Albert, 1994; Boje et al., 1997; Mattiucci and Nascetti, 2007, 2008; Karpiej et al., 2013). *P. bulbosa* has also been documented in the body cavity of Greenland halibut from the Barents Sea (Karpiej et al., 2013) and from the Davis Inlet (Paggi et al., 1991). Three species of third-stage larvae belonging to the *Anisakidae* family were detected in the current study; *A. simplex s. s*. was found in all examined organs. *C. osculatum s. s.* was identified in the body cavity, while *C. osculatum* A and B were found both in belly flaps, body cavities and liver. *P. bulbosa* was only isolated from the body cavity of one fish and the livers of three fish, which is consistent with the sporadic occurrence previously reported (Paggi et al., 1991; Karpiej et al., 2013), and probably due to the fact that *P. bulbosa* mainly infects benthic species – whereas Greenland halibut is benthopelagic with a preference for deep waters. It is noteworthy that *P. decipiens* was not found. This may partly be explained by the fact that the present study included molecular identification, which provides a more precise identification compared to morphological identification methods applied in some of the earlier studies. Alternatively, this particular species may be so rare that much larger samples should be examined in order to find a positive *P. decipiens* sample.

To the best of our knowledge, only two studies on Greenland halibut included examination of the fillets, although no details on the fillet distribution were included (Wierzbicka, 1992; Boje et al., 1997). Boje et al. (1997) investigated the prevalence and intensity of *A. simplex* infection in the fillets and body cavity of Greenland halibut from different locations around Greenland. Prevalence and intensity of *A. simplex* were reported to be 20 % and 1.95 ± 0.65 in fish from the Davis Strait (NAFO Subarea 1C/1D); 15 % and 1.9 ± 0.1 in fish from Uummannaq (NAFO Subarea 1A); and 72 % and 4.2 ± 2.9 in fish from the Denmark Strait (ICES 14b). The infection parameters presented by Boje et al. (1997) reflect a combined parasite burden of both the alimentary tract (esophagus, pyloric caeca, and intestine), while the current study investigated separately the precisely defined subsections of the musculature and organs. Additionally, the combination of UV-press and molecular identification is regarded more sensitive compared to older, classical approaches, which could affect both detection rate and species determination. However, as only a subsample of third-stage larvae was subject to molecular identification in the present study, it cannot be ruled out that single specimens of other species were not present.

In the present study, we noticed a higher occurrence of anisakid larvae in the main fillet and belly flaps of fish caught in E and SW compared to NW. Although a clear, statistically safe differentiation of the geographic groups was not possible due to the relatively low number of fish from some of the sampled zones, the observation resembles previous results (Boje et al., 1997). This might reflect that host availability and environmental conditions may be more favorable for larval transmission, hatch time and survival in E and SW (Kuhn et al., 2011, 2013); Several definitive, intermediate and/or paratenic hosts for *A. simplex* s. s. inhabit the waters around Greenland, although anisakid infection rates in Greenlandic habitats have been scarcely investigated (Brattey and Ni, 1992; Ólafsdóttir and Hauksson, 1998; Ramilo et al., 2024; Kumas et al., 2025). While ice-dependent seal species can be found across east and northwest regions, an abundance has been reported in East Greenland during ice seasons, and species such as harp seals are restricted to Southwest and East Greenland (Andersen et al., 2014; Hamilton et al., 2021; ICES, 2024a, ICES, 2024b). Additionally, migratory whale species are numerous in Southwest and East Greenland during summer (Heide-Jorgensen et al., 2007; Hansen et al., 2018).

Surrounding ocean currents and seasonal prey influx around southern and eastern Greenland likely add to this effect (Michalsen et al., 1998; Knutsen et al., 2007; Heide-Jørgensen et al., 2023). Diet composition impacts parasite burden, and the availability and abundance of paratenic and intermediate hosts can be subject to geographic and seasonal variation (Pedersen and Riget, 1993). While certain prey items such as northern shrimp (*Pandalus borealis*) and redfish (*Sebastes* spp.) are found in all regions, seasonal influx of prey can contribute to diet variation and parasite transmission. Studies from east Greenland have shown that fish and cephalopods dominate the diet of larger Greenland halibut (Michalsen et al., 1998; Vollen et al., 2004; Woll and Gundersen, 2004; Solmundsson, 2007), while main prey items on the western side in the Davis strait include northern shrimp, redfish, Arctic cod (*Boreogadus aida*) as well as other Greenland halibut (Pedersen and Riget, 1993; Anker Pedersen, 1994; Jørgensen, 1997b; Orr and Bowering, 1997). A study also found capelin (*Mallotus villosus*) to be more abundant in the stomachs of Greenland halibut from Southwest Greenland compared to the east (Woll and Gundersen, 2004). We are not aware of any studies investigating the diet of halibut from more northern waters off of Greenland, but studies on Greenland halibut from the Canadian Beaufort Sea and western Baffin Bay highlight Arctic cod as an important diet contributor (Giraldo et al., 2018; Pedro et al., 2023). While *A. simplex* has been reported to infect most fish species that Greenland halibut prey on (Margolis and Arthur, 1979; Marianne, 1993), its prevalence in northern shrimp, which is abundant in the Davis strait, is less elucidated but appears to be low (Shiraki et al., 1976; Smith, 1983). On top of that, the role of seasonal sea ice formation should not be neglected, and receding ice cover and freezing duration could increase host activity and larval transmission, and possibly expand the parasite's geographic range (Rokicki, 2009; Van Hemert et al., 2023; Mastick et al., 2024).

A higher abundance of *A. simplex* s. s. in Greenland halibut has been coupled with an offshore pelagic water habitat (Højgaard, 1998; Karpiej et al., 2013), and distribution of different parasite species may vary between offshore and inshore populations (Boje et al., 1997). Future studies should explore geographical differences in parasite burden further, and investigate the potential of using *A. simplex* s. s., and possibly *C. osculatum* and *P. bulbosa*, to differentiate halibut stocks, as previously suggested by other authors (Boje et al., 1997). Systematic monitoring of these parasites could help determine and track annual and seasonal activity of definitive host species in important fishing zones (Mackenzie, 2002; Marcogliese, 2005; Thompson et al., 2005), pollution (Sures, 2004) and climate change (Poulin, 2006; Brooks and Hoberg, 2007). Finally, it would aid continuous assessment of the zoonotic potential associated with consumption of these species (EFSA, 2010; Klimpel and Palm, 2011).

Parasite burden is usually a function of age and primary prey items (Boily and Marcogliese, 1995; Poulin, 2000; Nielsen and Andersen, 2001; Link and Garrison, 2002; Zhu et al., 2002), but in the current study, infection parameters seemed to be independent of body size or estimated age, except for a moderate positive correlation between liver parasitism and host size (Fig. 2b). This aligns with the findings of Boje et al. (1997) in their study of helminth parasites in Greenland halibut from six different Greenlandic fishing grounds (Boje et al., 1997), but contrasts the results of other studies, which have found the number of parasites to be directly proportional to the size of Greenland halibut caught in the Barents Sea and off the Labrador Shelf (Wierzbicka, 1991a; Karpiej et al., 2013). These differences could be related to geographical variations in definitive host abundance, intermediate hosts and feeding ecology, or attributed to enhanced fish immunity and infection clearance with increasing size (Priebe et al., 1991; Mladineo et al., 2011). Alternatively, the sample size or parasite count in the current study might simply have been too small to reveal such associations, with too few fish in the larger size categories. Correlation relies on variability to detect relationships, and in cases where most fish are not infected, as in the present study, it is difficult to show meaningful patterns.

*A. simplex s. s.*, the main pathogen associated with anisakidosis and anisakiasis, was identified as the dominating species in all investigated organs in the current study. Several commercial fish species with a circumpolar distribution can act as paratenic hosts of this parasite, including Atlantic cod (*Gadus morhua*) and Atlantic salmon (*Salmo salar*). Compared to these other economically valuable species, the parasite burden documented in the present study appears somewhat low (Strømnes and Andersen, 1998; Mouritsen et al., 2010; Gay et al., 2018; Kent et al., 2020; Severin et al., 2020), and the results demonstrate that infection with zoonotic third-stage anisakid nematode larvae predominantly occur in the belly flaps, body cavity and liver of Greenland halibut. The belly flaps are routinely discarded during industrial processing of halibut for fillets or loin portions, which likely reduces the presence of zoonotic anisakid larvae in the final product. Moreover, whole fish exported to overseas markets are subjected to prolonged freezing at temperatures and durations sufficient to inactivate larvae (−20 °C, or below, for days to weeks). As for non-industrial home preparation of freshly caught whole halibut, careful gutting and rinsing, along with removal of the belly flaps, is advised to reduce the risk of zoonotic transmission. To further avoid infection, adequate preparation either by heating or freezing in accordance with official guidelines is recommended (EFSA, 2010; EFSA et al., 2018; EFSA et al., 2024).

Lastly, high larval counts in the liver have been linked to high fillet and belly flaps parasite load in redfish (Klapper et al., 2015). While we did not find a correlation between visceral larvae count and fillet parasitism, we did find a moderately strong positive correlation between liver parasitism and belly flap parasitism (see subsection 4.2). While a total parasitological examination of the entire fish organism provides deeper insight, it is worth investigating whether the liver infection is a relevant and useful indicator of fillet parasitism in Greenland halibut, for example to estimate and prioritize the extent of manual processing of certain fish products, as discussed in the study by Klapper et al. (2015).

### 5.2 Associations between parasite burden, host biometrics and MHS

Fish affected by MHS carried a higher number of third-stage larvae in the belly flaps and liver. This difference was driven primarily by an over dispersed distribution (high parasite occurrence in a few individuals), and we found no statistical correlations between other parasite parameters and MHS. However, Greenland halibut affected by MHS showed a significantly higher GSI and were more frequently in mature or spawning stages compared to unaffected individuals. At the same time, these fish exhibited lower condition factor, suggesting compromised somatic condition. Although no correlations between reproductive status and parasite burden were established in the current study, such relationships have been documented in other species (Sufi et al., 1980; Skarstein and Folstad, 1996; Campbell et al., 2021).

While it is less likely that MHS can be attributed to infection with third-stage larvae, these patterns might reflect a possible link between MHS and chronic parasitic infections, which may need larger samples to reveal. A lower condition factor is often associated with long-term physiological stress, poor nutritional status, or sustained parasitism – all of which might contribute to or result from MHS (Severin and Buchmann, 2024). Conversely, the elevated GSI and greater proportion of spawning individuals among MHS-affected fish could indicate a terminal investment strategy, where fish experiencing chronic stress, either due to low nutrient intake and/or parasitism, allocate more resources to reproduction at the expense of somatic maintenance. Such a connection might be evident if MHS severity could have been factored in the calculation, but unfortunately our sample was too small for that. Moreover, QIM scores and gall bladder fullness differed significantly between normal fish and fish affected by MHS. This may be due to lowered food intake or indirectly indicate altered health status (McCormick and Podoliak, 1984; Robb, 1992). Previous investigations on MHS in Greenland halibut have indicated a difference in diet composition between fish affected and unaffected by MHS (Severin et al., 2025a, 2025b). The findings from the present study further suggest that reproductive biology and parasitism also factor into the equation, contribute to a deeper understanding of host-parasite interactions and MHS.

The absence of significant pathological signs at necropsy (except in one severely affected individual) and the moderate strength of correlations suggest that the relationship between MHS and parasite burden may be subtle or cumulative rather than acute. This aligns with findings from other chronic host-parasite systems, where physiological impacts are often not associated with overt lesions but still influence host performance over time (Umberger et al., 2013; Binning et al., 2017). Age and sex did not appear to be associated with MHS. However, neither thin-sectioning or bomb radiocarbon analysis was performed to validate the readings (Treble et al., 2008; Dwyer et al., 2016; Badaev et al., 2023), and the age estimates in the current study should therefore be considered with caution.

## 6 Conclusion

In the current study, three anisakid species were identified: *A. simplex* s. s., *C. osculatum* (subtype A and B), and *P. bulbosa*. The parasites were found mainly in the belly flap, body cavity and liver, and less frequently in the main fillet.

The study also revealed a complex, potentially indirect link between MHS and parasitism: MHS-affected individuals exhibited higher infection levels of the belly flaps and liver, in addition to elevated GSI, a higher frequency of mature or spawning stages, and reduced condition factor. These physiological patterns may suggest an investment strategy where stressed fish prioritize reproduction over somatic maintenance, possibly due to poor nutrition or low-level parasitism. Larger studies are needed to further clarify the physiological pathways linking MHS, parasitism, and reproductive strategies, and expanded sample sizes might reveal relationships that were not evident in the current study.

While consumption of raw or undercooked wild fish products poses a risk of contracting zoonotic nematodes, our findings indicate that accidental ingestion of third-stage anisakid larvae can be markedly reduced by discarding the belly flaps and visceral organs. This procedure is followed in the processing industry and extends to non-commercial and traditional ways of preparing Greenland halibut.

For future research directions, investigations across seasons and fishing grounds could elucidate whether *A. simplex* s. s. prevalence and abundance – or other parasite species with biomarker potential – might be used to differentiate fish stocks and monitor ecosystem alterations, parasite-host relationships and infection burden between stock complexes (MacKenzie, 1987; Arthur and Albert, 1994; Boje et al., 1997; Mackenzie, 2002). More information about differences in parasite parameters from different fishing locations could be applied in risk assessment of fish products related to place of capture.

The results presented here contribute with updated information on the parasite fauna and biology of Greenland halibut. The findings may aid future ecosystem and fish stock monitoring, and inform safe handling practices for both commercial and traditional fish consumption.

## CRediT authorship contribution statement

**Natacha L. Severin:** Writing – review & editing, Writing – original draft, Visualization, Project administration, Methodology, Investigation, Funding acquisition, Formal analysis, Data curation, Conceptualization. **Andrea K. Bisbjerg:** Writing – review & editing, Validation, Investigation, Formal analysis. **Kitt U. Ristinge:** Writing – review & editing, Validation, Investigation, Formal analysis. **Kaan Kumas:** Writing – review & editing, Validation, Investigation, Formal analysis. **Liliana I. Ferrão:** Writing – review & editing, Validation, Investigation, Formal analysis. **Per W. Kania:** Writing – review & editing, Visualization, Validation, Supervision, Software, Resources, Methodology, Funding acquisition, Formal analysis, Data curation, Conceptualization. **Kurt Buchmann:** Writing – review & editing, Validation, Supervision, Resources, Methodology, Investigation, Funding acquisition, Formal analysis, Conceptualization.

## Funding

The Greenland Research Council (*Nunatsinni Ilisimatusarnermik Siunnersuisoqatigiit*) funded the study via the PhD and Postdoctoral Stipends Program (grant no. 80.044). The grant is constituted by funds from The Danish State's Funds for Arctic Research, Bank of Greenland Business Fund and Royal Greenland A/S.

The Ministry of Food, Agriculture and Fisheries of Denmark funded the study via the OPTIKVAL project (program GUDP, grant no. 34009-22-2100).

## Declaration of competing interest

The authors declare that they have no known competing financial interests or personal relationships that could have appeared to influence the work reported in this paper.
