# Supplementary material for: Zoonotic nematode larvae in Greenland halibut (Reinhardtius hippoglossoides) from Greenlandic waters: Occurrence, fillet distribution and association with Mushy halibut syndrome
Source: Int J Parasitol Parasites Wildl. 2025 Dec 24;29:101181. doi: 10.1016/j.ijppaw.2025.101181 (PMC12796938; doi:10.1016/j.ijppaw.2025.101181)
Supplement: Multimedia component 2 [file mmc2.docx]

**Supplementary material 2: Necropsy Protocols**

**Article information**

**Title:** Zoonotic Nematode Larvae in Greenland halibut (*Reinhardtius hippoglossoides*) from Greenlandic waters: Prevalence, Fillet Distribution Association with Mushy Halibut Syndrome

**Journal:** International Journal for Parasitology: Parasites and Wildlife

**Authors:** Natacha L. Severin ^a^ ^[[1]](#footnote-1)^, Andrea K. Bisbjerg^a^, Kitt U. Ristinge^a^, Kaan Kumas ^a^, Liliana Ferrão ^b^, Per W. Kania ^a^, Kurt Buchmann ^a^

**Affiliations:**

^a^ Laboratory of Aquatic Pathobiology (AQUA), Section for Parasitology and Pathobiology, Department of Veterinary and Animal Sciences, Faculty of Health and Medical Sciences, University of Copenhagen, Stigbøjlen 7, 1870, Frederiksberg C, Denmark

^b^ Department of Animal Sciences and Aquatic Ecology, Ghent University, Coupure Links 653 – block F, B-9000 Gent, Belgium

**Correspondence:** Natacha L. Severin, [nls@sund.ku.dk](mailto:nls@sund.ku.dk)

**Protocol for Necropsy (sample: n = 104)**

| **Date (DD/MM/YY)** |  |
| --- | --- |
| **Fish ID** |  |

1. Exterior inspection

**Whole fish length:** ____________ cm / **Thawed** **whole fish weight:** ____________ kg

**Fish thickness:_** ______________ cm / **Fish width:** _________________________ cm

|  | **Inspection** | | **Injuries/lesions** | | **Visible parasitism** | | **Comments** |
| --- | --- | --- | --- | --- | --- | --- | --- |
| **Overall condition** | Good |  | Yes |  | Yes |  |  |
|  | Average |  | No |  | No |  |  |
|  | Below average |  |  |  |  |  |  |
|  | Thin to cachexic |  |  |  |  |  |  |
| **Integument** | Tight skin |  | Yes |  | Yes |  |  |
|  | Loose/wrinkly skin |  | No |  | No |  |  |
|  | Discoloration |  |  |  |  |  |  |
|  | Petechiae/ Ecchymoses |  |  |  |  |  |  |
|  | Net marks |  |  |  |  |  |  |

1. Necropsy

**Fish weight post evisceration:** ____________ kg

| **Belly flap** | **Appearance** | | **Storage mode** | **Weight (g)** | |
| --- | --- | --- | --- | --- | --- |
| *Visceral organs (inspection in situ) and body cavity* | Normal |  |  |  | |
|  | Abnormal |  |  |  |  |
|  | Parasitism |  |  |  |  |
|  | Discoloration |  |  |  |  |
| *Liver*  *(excl. gallbladder and bile duct)* | Normal |  | Pack individually for parasitology |  | |
|  | Abnormal |  |  |  |  |
|  | Parasitism |  |  |  |  |
| *Gonads* | Immature |  | Pack individually for inspection | Sex | |
|  | Maturing |  |  | ♂ |  |
|  | Spawning |  |  | ♀ |  |
|  | Spent |  |  |  |  |
| *Skeletal muscle (fillets)* | Firm |  | Pack individually for parasitology | Divide each fillet into dorsal and ventral belly flaps, and dorsal and ventral anterior, medial and posterior fillet parts | |
|  | Soft |  |  |  |  |
|  | MHS |  |  |  |  |
|  | Visible parasitism |  |  |  |  |

1. Fillets

**Fillet weight:** __________ g

| **Assessment of MHS** | | **Appearance** | **Consistency** | **Other** | |
| --- | --- | --- | --- | --- | --- |
| Normal |  | *Shiny white to slight opaque* | *Firm, normal elasticity*  *Little to no moisture release upon manual pressure* | Reddish |  |
| MHS |  | *Glassy or opaque to translucent* | *Soft with decreased elasticity*  *Slippery, gelatinous and fragile*  *Moisture release upon manual pressure* | Blood stains |  |
|  |  |  |  | Yellowish |  |
|  |  |  |  | Greenish |  |
|  |  |  |  | Grey |  |

1. Maturity staging

Overview of criteria used in maturity staging of 104 Greenland halibut, as well as corresponding index value used for statistical calculations. Modified after Albert et al. (2001).

| **Maturity stage** | **Index** | **Criteria** |
| --- | --- | --- |
| Immature | 0 | Colourless small gonads |
| Maturing | 1 | Larger gonads with visible oocyte development and whitening of the testes |
| Spawning | 2 | Running gonads. Fully developed oocytes and milt, which can be released by applying light pressure on the abdomen |
| Spent | 3 | Slack gonads which may contain residual oocytes or milt |

**Protocol for Extended Necropsy (subsample: n = 55)**

| **Date (DD/MM/YY)** |  |
| --- | --- |
| **Fish ID** |  |

1. Exterior inspection

**Whole fish length:** ____________ cm / **Thawed** **whole fish weight:** ____________ kg

**Fish thickness:_** ______________ cm / **Fish width:** _________________________ cm

|  | **Inspection** | | **Injuries/lesions** | | **Visible parasitism** | | **Comments** |
| --- | --- | --- | --- | --- | --- | --- | --- |
| **Overall condition** | Good |  | Yes |  | Yes |  |  |
|  | Average |  | No |  | No |  |  |
|  | Below average |  |  |  |  |  |  |
|  | Thin to cachexic |  |  |  |  |  |  |
| **Integument** | Tight skin |  | Yes |  | Yes |  |  |
|  | Loose/wrinkly skin |  | No |  | No |  |  |
|  | Discoloration |  |  |  |  |  |  |
|  | Petechiae/ Ecchymoses |  |  |  |  |  |  |
|  | Net marks |  |  |  |  |  |  |
| **Fins** |  | | Yes |  | Yes |  |  |
|  |  |  | No |  | No |  |  |
| **Oral cavity** | Regurgitation |  | Yes |  | Yes |  |  |
|  | Other |  | No |  | No |  |  |

1. QIM (López-Gárcia et al. 2013)

| **Quality parameters** | **Description** | **Points** | **Points** |
| --- | --- | --- | --- |
| *Appearance skin (D_1_)* | Very bright  Bright  Dull | 0  1  2 |  |
| *Flesh elasticity (D_2_)* | Very firm, elastic (finger mark disappears rapidly)  Firm (finger leaves mark over 3 s)  Soft (finger print remains) | 0  1  2 |  |
| **Eyes** | | | |
| *Shape (D_3_)* | Convex  Flat  Concave | 0  1  2 |  |
| *Clarity (D_4_)* | Black, silver rim around the pupil  Matt, without silver rim  Milky | 0  1  2 |  |
| **Gills** | | | |
| *Color (D_5_)* | Brick red  Pale red, pinkish  Greenish brown | 0  1  2 |  |
| **Body and flesh** | | | |
| *Mucus (D_6_)* | Absent Clear Yellowish, slightly clotted Brown, clotted | 0  1  2  3 |  |
| *Odour (D_7_)* | Sea, seaweedy Neutral Rancid, acid, sour Rotten | 0  1  2  3 |  |
| *Colour of flesh (D_8_)* | Ivory, translucent  Slightly yellowish, opaque  Greenish yellow, opaque | 0  1  2 |  |
| **Peritoneum and bone** | | | |
| *Blood in bone (D_9_)* | Bright red, pinkish, not present  Red brown  Brown | 0  1  2 |  |
| *Guts (D_10_)* | Whole, bright pink  Whole, slightly brown  Mass without structure, darker brown | 0  1  2 |  |
| *Peritoneum (D_11_)* | Very attached  Easily separable  Disintegrated | 0  1  2 |  |
| **Quality index** | | **24** |  |

*QI = 0 indicates the highest/freshest quality.*

1. Necropsy

**Fish weight post evisceration:** ____________ kg

| **Exterior** | **Target organ or structure** | | **Storage mode** | **Notes** | |
| --- | --- | --- | --- | --- | --- |
| *Head* | Otolith | | Rinse and store in paper envelopes |  | |
| *Gills* | Arch and filaments | | Clippings in 10% formalin 24h, then transfer to 70% EtOH | Only if macroscopic signs of disease | |
| **Belly flap** | **Appearance** | | **Storage mode** | **Weight (g)** | |
| *Visceral organs (inspection in situ) and body cavity* | Normal |  |  |  | |
|  | Abnormal |  |  |  |  |
|  | Parasitism |  |  |  |  |
| *Ventricle* | Empty |  | Bag and store on ice for immediate stomach content analysis  Discard if regurgitation |  | |
|  | Very little content |  |  |  |  |
|  | Some content |  |  |  |  |
|  | Full |  |  |  |  |
|  | Very full (stretched) |  |  |  |  |
|  | Regurgitation |  |  |  |  |
| *Intestines* | Empty |  |  |  | |
|  | Some content |  |  |  |  |
|  | Full |  |  |  |  |
| *Spleen* | Normal |  |  |  | |
|  | Enlarged |  |  |  |  |
|  | Discoloration |  |  |  |  |
| *Liver*  *(excl. gallbladder and bile duct)* | Normal |  | Pack individually for parasitology |  | |
|  | Abnormal |  |  |  |  |
|  | Parasitism |  |  |  |  |
| *Gallbladder* | Empty |  |  |  | |
|  | Some content |  |  |  |  |
|  | Full |  |  |  |  |
| *Bile color* | Pale yellow-green |  | Aspirate with syringe to assess color |  | |
|  | Dark yellow/brown |  |  |  |  |
|  | Dark green/blue |  |  |  |  |
| *Heart* | Normal |  | Pack individually for parasitology |  | |
|  | Abnormal |  |  |  |  |
|  | Parasitism |  |  |  |  |
| *Gonads* | Immature |  | Pack individually for inspection | Sex | |
|  | Maturing |  |  | ♂ |  |
|  | Running/spawning |  |  | ♀ |  |
|  | Spent |  |  |  |  |
| *Skeletal muscle (fillets)* | Firm |  | Pack individually for parasitology | Divide each fillet into dorsal and ventral belly flaps, and dorsal and ventral anterior, medial and posterior fillet parts | |
|  | Soft |  |  |  |  |
|  | MHS |  |  |  |  |
|  | Visible parasitism |  |  |  |  |

1. Fillets

**Fillet weight:** __________ g

| **Assessment of MHS** | | **Appearance** | **Consistency** | **Other** | |
| --- | --- | --- | --- | --- | --- |
| Normal |  | *Shiny white to slight opaque* | *Firm, normal elasticity*  *Little to no moisture release upon manual pressure* | Reddish |  |
| MHS |  | *Glassy or opaque to translucent* | *Soft with decreased elasticity*  *Slippery, gelatinous and fragile*  *Moisture release upon manual pressure* | Blood stains |  |
|  |  |  |  | Yellowish |  |
|  |  |  |  | Greenish |  |
|  |  |  |  | Grey |  |

1. Maturity staging

Overview of criteria used in maturity staging of 104 Greenland halibut, as well as corresponding index value used for statistical calculations. Modified after Albert et al. (2001).

| **Maturity stage** | **Index** | **Criteria** |
| --- | --- | --- |
| Immature | 0 | Colourless small gonads |
| Maturing | 1 | Larger gonads with visible oocyte development and whitening of the testes |
| Spawning | 2 | Running gonads. Fully developed oocytes and milt, which can be released by applying light pressure on the abdomen |
| Spent | 3 | Slack gonads which may contain residual oocytes or milt |

1. [↑](#footnote-ref-1)
